# A novel mutation in FK506 binding protein-like (*FKBPL*) causes male infertility

**DOI:** 10.3325/cmj.2021.62.227

**Published:** 2021-06

**Authors:** Derya Akyuz Sengun, Esra Guzel Tanoglu, Hakan Ulucan

**Affiliations:** 1 Department of Medical Genetics, Istanbul University Cerrahpasa Medical School, Istanbul, Turkey; 2 Department of Molecular Biology and Genetics, University of Health Sciences, Istanbul, Turkey

## Abstract

**Aim:**

To perform a mutation analysis of FK506 binding protein-like (*FKBPL*) in patients with azoospermia.

**Methods:**

DNA samples were isolated from the peripheral blood of 30 azoospermic male patients with normal 46 XY karyotype and 10 healthy controls. Multiplex polymerase chain reaction assays were used to evaluate Y microdeletions, and the patients without deletions were further analyzed. Sanger sequencing was used for mutation analysis.

**Results:**

A heterozygous adenine to guanine substitution was observed at position c.28 (c.28A>G) (one patient), guanine to adenine substitution at c.90 (c.90G>A) (three patients), and a novel insertion mutation of TCTCATAAGTCT at c. 229_240dup (two patients), all in *FKBPL* exon 2. Furthermore, four different benign variants were observed in the same gene.

**Conclusion:**

Our study supports the literature on the etiologic effects of changes on autosomal chromosomes and highlights the importance of molecular analysis of all known and unknown genes that could be involved in male sexual development and function.

Genetic alterations are implicated in the pathogenesis of male infertility in 10%-15% of patients (1). The most common cause of azoospermia are microdeletions of the AZF region on the Y chromosome (2,3). Only a small proportion of other pathogenic variants in azoospermia cases have been identified, and other genetic causes need to be explored (4). In most infertility clinics, chromosome Y microdeletion analysis is the last step in molecular male infertility diagnostics (5). However, this analysis alone is insufficient since male infertility is related to complex genetic alterations.

To date, many genes have been investigated for their effect on spermatogenesis. As a result, many pathogenic and normal variants that affect protein expression, structure, and function in spermatogenesis have been identified and associated with azoospermia infertility. However, the studies are still insufficient in terms of determining the diagnostic factors, and infertility remains a major problem worldwide. Genetic alterations of autosomal genes could be emerging causes of infertility in azoospermic men negative for Y microdeletions.

Linkage analysis and clustering of chromosomal breakpoints in infertile men showed that FK506-binding protein like (*FKBPL*) gene pathogenic variants play an important role in azoospermia (6,7). *FKBPL*, a member of the *FKBP* family (DIR1, WISp39), was first described in 1999 and is chromosomally localized at the position 6p21.3. It is strongly expressed in the testis, including the Sertoli and Leydig cells (8,9). Pathogenic variants in *FKBPL* cause male infertility by disrupting the androgen receptor (AR) signal. *FKBPL* has been reported to increase the transactivation of AR-responsive genes.

The aim of this study was to investigate whether the pathogenic variant analysis of *FKBPL* could be used as a routine test in azoospermic men negative for Y microdeletions, since autosomal genes are not routinely tested.

## Materials and methods

### Blood samples

Participants were selected among male patients who were diagnosed with azoospermia in the Department of Medical Genetics, Cerrahpaşa Medical Faculty of Istanbul University, from 2012 to 2015. After patients underwent detailed medical history-taking and a physical examination, blood samples were obtained, and cytogenetic and molecular investigations were performed. The study enrolled patients with normal karyotype who had only primary infertility. Individuals with Y microdeletion or chromosomal anomaly and individuals not diagnosed with azoospermia were excluded. Finally, 30 men with azoospermia and 10 men with normospermia were included. The control group was selected from healthy staff members who have children. All patients and controls signed an informed consent form, and the study was approved by the Ethics Committee of the Istanbul University Cerrahpasa Medical Faculty (239656).

### Total DNA isolation

DNA was isolated according to the Invitrogen DNA isolation kit (Invitrogen, Waltham, MA, USA) protocol, and DNA samples were stored at -20 °C. DNA concentration and purity were assessed with the NanoDrop ND-2000c (Thermo Fisher Inc., Dreieich, Germany) spectrophotometer.

### Y microdeletion analysis

AB Analitica AZF mix kit was used for the Y microdeletion analysis of AZFa, AZFb, and AZFc regions. Multiplex polymerase chain reaction (PCR) was performed using three separate mix tubes to screen for deletions in AZF loci. The primers from specific regions were as follows: Oligomix 1: ZFY/ZFX, SRY, sY254, sY86, sY127, sY255; Oligomix 2: ZFY/ZFX, SRY, sY95, sY117, sY125; Oligomix 3: DBY, ZFY/ZFX, SRY, sY84, sY134, DFFRY. After PCR, the samples were run on a 3% agarose gel, and imaging was performed.

### *FKBPL* gene analysis

For the *FKBPL* gene, primers were designed with Ensembl, Primer 3, and NCBI Primer Blast programs. PCR was performed with the sequence of the designed primer. The samples were amplified using the following primers: forward 5′CGCGCCAAATTCTGTTCGAT3′, reverse 5′GCTTCTATGTGTCCTCGGAGA‘3, and internal primer 5′GTGCTGACCTGAACCTGG‘3. The PCR mixture contained 12.5 μL Y Microdeletion Multiplex PCR Mix (AB Analitica AZF-MX V2.0, Padua, Italy), 10.5 μL distilled water, 0.5 μM forward primer, 0.5 μM reverse primer, and 1 μL (for 100 ng) of DNA. After preincubation at 95 °C for 5 minutes, 35 PCR cycles were performed at 95 °C for 30 seconds, at 60 °C for 90 seconds, and at 72 °C for 90 seconds. The reaction was extended at 68 °C for 10 minutes.

### Sanger DNA sequencing

One microliter of Exonuclease I of ExoSap (Affymetrix, ABD, Thermo Fisher Inc.) and 2.5 μL of PCR sample were mixed in a separate tube and placed in a PCR thermal cycler for 15 minutes at 37 °C and at 80 °C at 15 minutes, for a total of 30 minutes. After this treatment, the PCR products were purified, and the resulting artifacts were removed with the QiaEX II DNA Purification Kit (Qiagen Laboratories Inc., Germantown, MD, USA). A sequencing PCR program was then applied to the tubes. The DNA sequencing reaction was carried out with BigDye, version 1.1 (Life Technologies, Darmstadt, Germany) using a 3500 Genetic Analyzer (Applied Biosystems, Waltham, MA, USA).

### Data analysis

Reference sequences obtained from the Ensembl database were used to compare the patients' DNA sequences. The sequences were analyzed with Codon Code Aligner Software 3.0 (CodonCode Corporation, Centerville, MA, USA) with the ENST00000375156.4 transcripts. Benign variant and mutation databases were searched to determine whether a base change was previously reported. The pathogenicity of unreported base changes was evaluated with *in silico* software: Sorting Intolerant From Tolerant (SIFT), Phyre2, Mutation Taster, and Polyphen2. To visualize the conformational changes of protein structure in wild-type and *FKBPL* mutants, we used Phyre2, an open-source Java viewer for chemical structures in D program.

## Results

**Figure 1:**
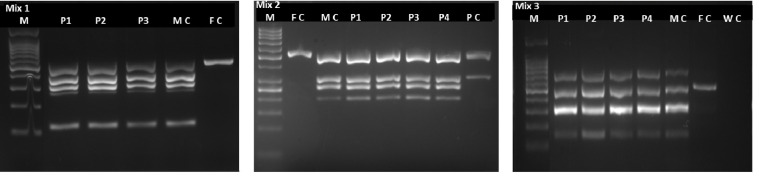
Representative gel images showing the positions of the multiplex primers for the detection of AZFa, AZFb, and AZFc regions in 3 (Mix 1) and 4 (Mix 2 and 3) patients. M –marker, FC – female control, MC – male control, WC – water control, and PC – positive control.

The patients' mean age was 35.7 ± 6.83 years and that of the controls was 37.5 ± 7.82 years. The purity of DNA isolated from blood ranged from 1.8 to 2. After appropriate DNA concentrations were obtained, deletion analysis was performed with the Y microdeletion method. A positive control, female control, male control, and water controls were used to ensure the study reliability. No deletions were detected in patients and controls (Figure 1).

The *FKBPL* variations in patients and controls, and c.-54C>T, c.28 A>G variation allele frequencies according to gnomAD software are shown in Table 1.

**Table 1:** The *FKBPL* variations in patients and controls, and c.-54C>T, c.28 A>G variation allele frequencies according to gnomAD software

Exon	Nucleotide change	Amino acid change	Allele frequency	Affected samples
**1**	c.190C>T	-		P2 P4 P7 P8 P9 P13 P15 P16 C1 C3 C4 C8 C10
**1**	c.175T>C	-		P14
**1**	c.83C>T	-		P15 P24 P25 P26 C8
**2**	c.-54C>T	-	6.27e-2	P10 P11 P12 P22 P26 P27 C10 (het) P6 (hom)
**2**	c.28A>G	p.Asn28Ser	5.81e-3	Missense mutation (P8)
**2**	c.90G>A	p.Ala41Thr		Missense mutation (P15 P24 P26)
2	**c. 229_240dup** **TCTCATAAGTCTins**	**Ser-His-Lys-Ser**		**Small-insertion (P9 P27)**

**Figure 2:**
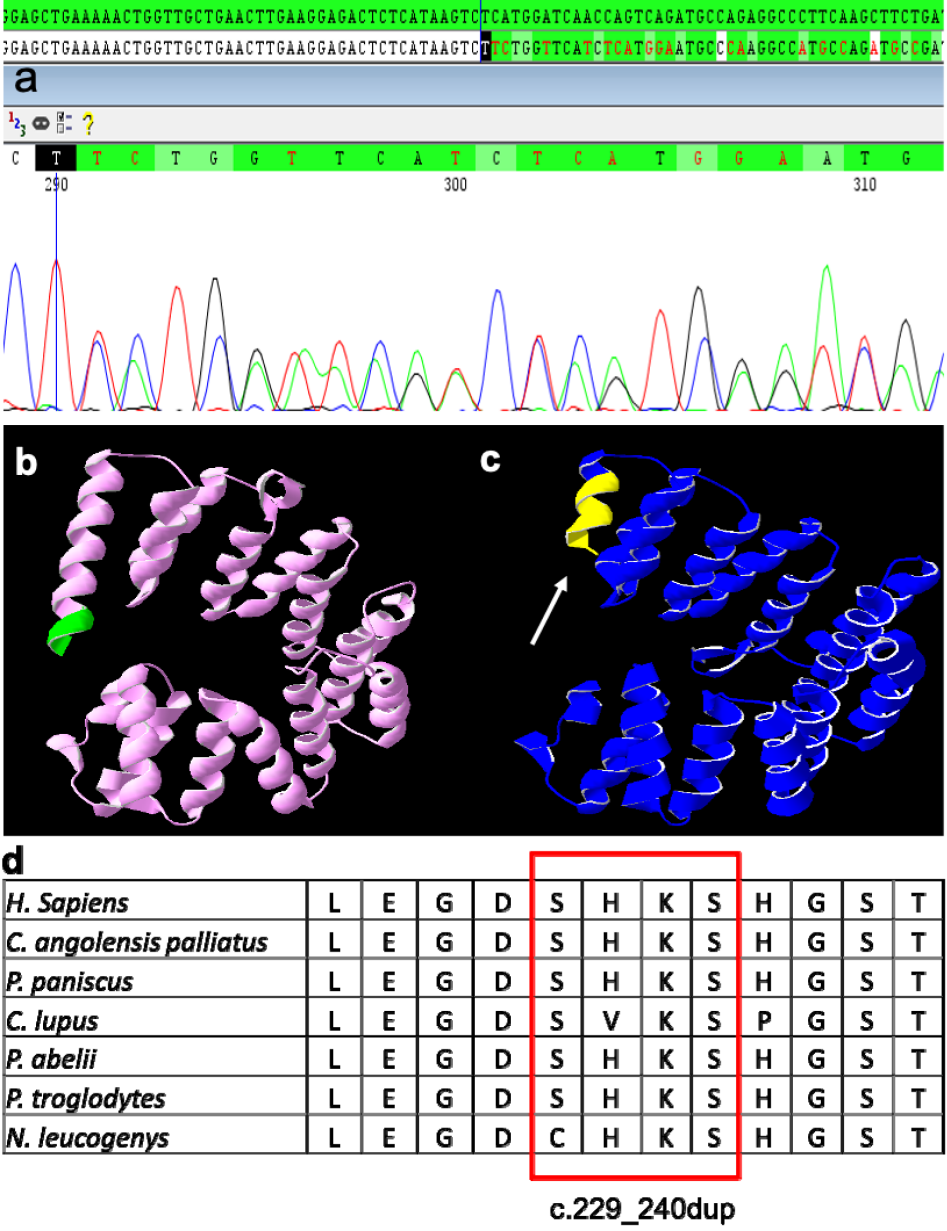
(**A**) Sanger sequencing: insertion mutation of TCTCATAAGTCT c.229_240dup in exon 2 of *FKBPL*. (**B**) Wild-type protein structure of *FKBPL.* (**C**) Mutant protein structure of *FKBPL* (the mutated region indicated by arrow). (**D**) The highly conserved amino acid sequence of interest throughout the species.

A missense mutation of c.90G>A was found in three patients, a missense mutation of c.28A>G in one patient, and an insertion of c. 229_240 in two patients (Figure 2A). According to the benign variant database, the other four single-base changes were benign variants.

*In silico* analysis of these missense variations showed that the insertion of TCTCATAAGTCT c. 229_240dup in exon 2 of *FKPBL* was the only mutation disrupting the three-dimensional structure of the FKPBL protein (Figure 2B and 2C).

The nucleotides that were duplicated for highly conserved amino acids among several species are shown in Figure 2D. This conservation of the wild-type protein structure among species also supports the notion that this insertion may disrupt the normal structure and function of FKPBL protein.

## Discussion

This study observed previously reported substitution mutations and a novel insertion mutation in *FKPBL* in patients with azoospermia.

Infertility affects about 15% of couples, and about 50% of primary infertility causes can be explained by the male factor (10). The most common genetic cause is Klinefelter syndrome, followed by deletions in the AZF region on the Y chromosome (11). However, studies on monogenetic causes are limited. Semen analyses, hormonal analyses, cytogenetic tests, and Y microdeletion tests are routinely performed in patients with primary infertility, and *CFTR* gene mutation tests are performed if obstructive azoospermia is suspected (12).

Recent studies have focused on non-gonosomal causes of male infertility. Mutations and variations in autosomal genes, which may affect spermatogenesis, have been shown to cause unexplained infertility (13). Among these autosomal genes, *FKBPL*, which is strongly associated with spermatogenesis, has been well studied to date. A previous study in a Japanese population reported that *FKBPL* was responsible for azoospermia (6).

Immunosuppressive FKBPs may act as chaperones to secure the stability of large steroid receptor complexes in the absence of a ligand (14). Androgens are the main steroid hormones that determine male phenotype characteristics. Since androgen activity is controlled by the *AR* gene, mutations in this gene have been proposed to impair spermatogenesis (15). *FKBP52* is an AR chaperone with demonstrated effects in experimental infertile mouse studies. However, while AR function is expected to decrease in the mouse testes without *FKBP52* function, observation of normal spermatogenesis has indicated the possibility that another gene, such as *FKBPL*, could be responsible for the dysfunction. It has been shown that *FKBPL* is more highly expressed in the testis, acts as an androgen receptor regulator, and may be associated with infertility (16,17).

Sunnotel et al (7) aimed to evaluate whether *FKBPL* mutations contribute to the azoospermic phenotype in mouse and human tissues. The authors detected a heterozygous missense mutation (A>G) at codon 28 of exon 2 of *FKBPL* (7). This was the first description of pathogenic alterations in *FKBPL* in azoospermic patients, indicating a potential role of this gene in AR-mediated signaling in the testis. The same mutation was also detected in a patient in our study. We also detected a heterozygous missense mutation (G>A) at codon 90 of exon 2 in other three patients. However, Polyphen2 and SIFT software programs showed that these two variations, defined as “benign” and “tolerable,” did not impair the protein function.

To the best of our knowledge, this is the first literature report of a novel small insertion TCTCATAAGTCT c.229_240dup in exon 2 of *FKBPL*. A simulation analysis performed with the Mutation Taster program suggested that the protein product formed might have caused the disease. Phyre2 program enabled us to compare the PDB files of the mutant and wild-type proteins in this region with those created by 3D-generated Jmol program, showing a disrupted protein structure. Conservation analysis of the sequenced locus also confirmed that this four-amino acid insertion might disrupt the normal structure and function of the FKPBL protein.

In conclusion, we found a novel four-amino acid insertion mutation of c.229_240dup in exon 2 as well as two missense mutations in the *FKBPL* gene, which may cause male infertility. As little as 5% of all azoospermic men have microdeletions on their Y chromosome, and no other molecular analyses are conducted as routine tests. Our study supports the literature findings on the etiologic effects of changes on autosomal chromosomes and confirms the importance of molecular analysis of all known genes that could be involved in male sexual development and function. Our next goal is searching for the unknown genetic origins of male infertility by using novel genome-wide scanning methods such as array-CGH, whole exome sequencing, and whole genome sequencing. Future studies could benefit from *in vivo* studies and functional analyses with an aim to develop routine biomarkers and better treatments of primary male infertility.
